# Research on the expression of elastin in the conjoint fascial sheath for the correction of severe unilateral congenital blepharoptosis

**DOI:** 10.1186/s12886-022-02469-w

**Published:** 2022-06-08

**Authors:** Zhaochuan Liu, Xin Jia, Runhui Pang, Huixing Wang, Junhu Shi, Ping Bai

**Affiliations:** 1 Department of Ophthalmology, Beijing Tongren Hospital, Beijing Ophthalmology and Visual Science Key Laboratory, Capital Medical University, Beijing, 100730 China; 2 Department of Ocular Plastic, Hebei Provincial Key Laboratory of Ophthalmology, Hebei Provincial Eye Institute, Hebei Eye Hospital, Xingtai, 054001 Hebei China

**Keywords:** Conjoint fascial sheath, Levator palpebrae muscle, Severe congenital blepharoptosis, Elastin, Immunofluorescent staining

## Abstract

**Background:**

To investigate the expression of elastin in the conjoint facial sheath (CFS) in patients with severe unilateral congenital blepharoptosis in different age groups.

**Methods:**

Twenty-seven cases of severe unilateral congenital blepharoptosis (27 eyes) were treated with CFS + LM complex suspension from January 2020 to July 2020. Within that sample, 9 patients were over 18 years old, 9 patients were 13 to 17 years old and 9 patients were 5 to 12 years old. CFS and LM specimens were collected during CFS + LM complex suspension surgery. In the CFS specimens, the elastic fibers were observed by Victoria Blue staining. The elastin expression levels of the three groups of specimens were determined and analyzed by immunofluorescent staining and Western blotting.

**Results:**

Victoria Blue staining showed that elastic fibers were abundant in CFS tissue. Moreover, immunofluorescent staining showed strong positive expression of elastin in the CFS and LM. Furthermore, in the child group, the Western blot results demonstrated that the expression of elastin was higher in the CFS than in the LM (*P* < 0.05). Additionally, the expression of elastin was significantly higher in the CFS of children than in that of adults or adolescents (*P* < 0.001).

**Conclusions:**

The CFS and LM are rich in elastic fibers and elastin, although elastin expression in the CFS decreases with age. Thus, it is feasible to apply CFS + LM complex suspension to cure severe unilateral congenital blepharoptosis.

**Supplementary Information:**

The online version contains supplementary material available at 10.1186/s12886-022-02469-w.

## Introduction

The conjoint fascial sheath (CFS) is a fibrous connective tissue of the levator palpebrea muscle and superior rectus, which is attached to the conjunctival fornix [1, 2]. Since 2002, CFS suspension has been applied to correct blepharoptosis; this procedure has a satisfactory curative effect and produces fewer complications than other procedures for blepharoptosis correction [3]. Recently, CFS + LM complex suspension has achieved better clinical effects than CFS suspension in the correction of severe blepharoptosis [4, 5]. Additionally, researchers have gradually begun to conduct anatomical and histological research on the CFS. Recent research has confirmed that the CFS is rich in collagen fibers and elastic fibers, while it lacks smooth muscle cells and skeletal muscle cells [1]. Elastic fibers and collagen fibers are key structures that enable tissues to maintain elasticity and withstand load-bearing stress, and elastin is the most important structural component of elastic fibers [6]. Therefore, the aim of this study was to investigate the expression of elastin in the CFS and LM of patients with severe unilateral congenital blepharoptosis and to further explore the histological structure of the CFS as well as the correlation between elasticity and age.

## Methods

### Patients

A total of 27 patients (27 eyes) with severe unilateral congenital blepharoptosis who were treated surgically from January 2020 to December 2020 underwent CFS + LM suspension. CFS and LM tissue specimens were collected during surgery. The patients were divided into an adult group (≥ 18 years old), an adolescent group (13–17 years old), and a child group (5–12 years old) according to their ages. The adult group consisted of 9 cases and the average age was 7.78 ± 1.99 years. The adolescent group consisted of 9 cases and the average age was 14.78 ± 1.39 years. The child group consisted of 9 cases and the average age was 23.67 ± 3.54 years. The specimens were cleaned immediately after being collected during the operation. One part was fixed with 10% formaldehyde solution, and the other part was stored in a refrigerator at -80 °C.

The inclusion criteria included patients with severe unilateral congenital blepharoptosis, patients without any eyelid surgery history, and patients with positive Bell’s phenomenon before surgery. The exclusion criteria were as follows: patients with contraindications for eyelid surgical intervention, patients with oculomotor nerve palsy, patients with immune diseases or connective tissue diseases, patients with systemic diseases, and patients with psychosis history.

The study adhered to the tenets of the Declaration of Helsinki for research involving human subjects, and all specimens were collected with the informed consent of the patients or family members, who signed an informed consent document. In addition, written informed consent for publication of their clinical details and clinical images was obtained from the patient or parent of the patient.

### Surgical technique and postoperative evaluation

**Fig. 1:**
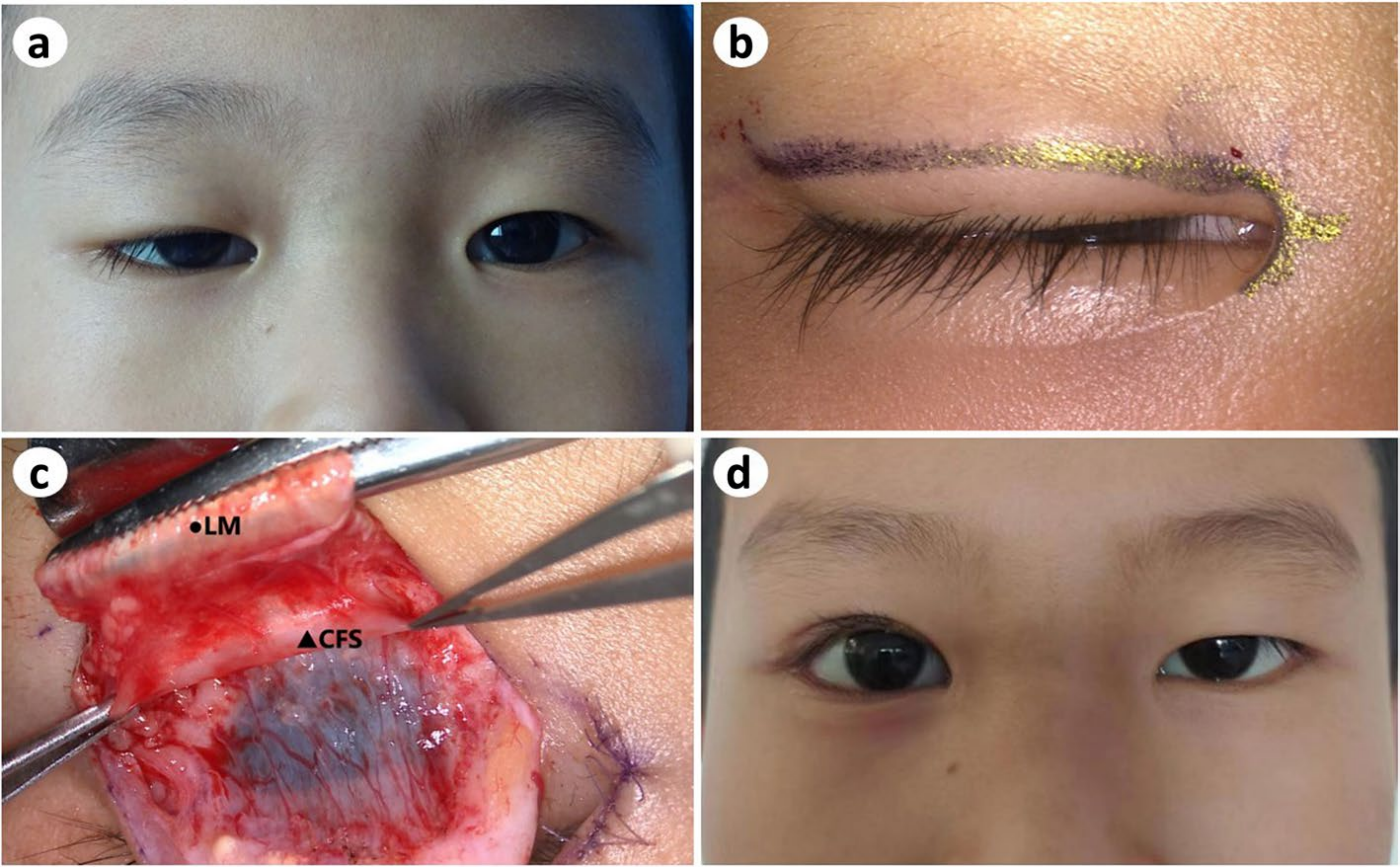
CFS + LM suspension to correct unilateral congenital blepharoptosis. **a**: Preoperative image of a five-year-old boy with congenital blepharoptosis OD; **b**: incision line labeled with methylene blue; **c**: exposure of CFS and LM; **d**: image of the patient twenty-two months after CFS + LM suspension

All CFS + LM suspension surgeries were performed by the same surgeon (P.B.). The surgical incision consisted of a double eyelid line incision; its course was marked in advance with methylene blue (Fig. 1B). The upper lid was anesthetized using ropivacaine plus 1:10,000 epinephrine. Fristly, cut the skin along the marked line and separete the subcutaneous tissue. Then, the orbicularis oculi was cut at a width of 2 mm before the tarsal plate. Cut the aponeurosis of the levator in the same length of incision parallel to the margin of the tarsal plate. The levator aponeurosis was carefully separated from the Müller muscle, leaving the Müller muscle and conjunctiva intact under the aponeurosis. The levator aponeurosis was detached from the Müller muscle and pulled approximately 4–5 mm over the fornix to expose the CFS. As is shown in Fig. 1C, the CFS is a sheath-like connective tissue whose thickness varies among individuals. The upper margin of the tarsus was suspended and fixed to the CFS + LM complex at a certain length according to patients' margin reflex distance 1 (MRD1). With reference to the position of the tarsus and pupil, U-shaped stitches of 5–0 absorbable thread were applied at the outer, intermediate, and inner positions. The patient was instructed to sit up to adjust the upper eyelid height to a satisfactory position, after which the dermis of the orbicularis oculi muscle was sutured to the tarsus using 6–0 nylon stitches, and the skin incision was closed with 6–0 nylon stitches which were removed one week after surgery. Additionally, in order to maintain symmetry, we consulted with the patients and their parents regarding whether to perform double plepharoplasty in the other eye or not. As can be seen in Fig. 1 A and D, the child’s parents decided to only undergo blepharoptosis correction on the affected eye and to receive blepharoplasty in the fellow eye when the child grew up.

The time points of follow-up were set as preoperatively, 1 month postoperatively, 3 months postoperatively and 6 months postoperatively. The curative effect of CFS + LM suspension was evaluated according to the following criteria [7]: (1) upper eyelid located 1 ~ 2 mm below the upper corneal limbus was considered to be well-corrected; (2) upper eyelid located > 2 mm below the upper corneal limbus was considered to be undercorrected; (3) upper eyelid located at or above the upper corneal limbus was considered to be overcorrected; and (4) no changes in the position of upper eyelid margin were considered to be recurrence.

### Histopathological evaluations

CFS and LM specimens were fixed with 10% formalin solution, embedded in paraffin, cut into 4-μm-thick sections (using a LEICA RM2155 microtome, Germany) and stained with a Victoria Blue staining kit (G1596, Solarbio, Beijing, China) for histopathological examination under a microscope (LEICA DM5000B microscope, Germany).

### Immunofluorescence

CFS and LM specimens from the same eye were removed from the refrigerator, embedded in liquid Optimal Cutting Temperature (OCT) compound at room temperature, and frozen until the OCT compound solidified. Frozen sections were cut at a thickness of 8 μm and air-dried for 30 min, and the boundaries were marked with a histochemical pen. The specimens were fixed in 4% paraformaldehyde solution for 30 min, immersed in PBS, soaked in 0.3% Triton X-100 for 10 min, and blocked with 1% BSA at room temperature for 1 h. After the blocking solution was discarded, the specimens were incubated with a rabbit anti-human elastin primary antibody (1:500, Abcam, USA) in a humidified chamber at 4 °C overnight, then immersed in PBS solution; next, they were incubated with a goat anti-rabbit IgG H + L (Alexa Fluor 488 conjugate) secondary antibody (1:1000, Abcam, USA) in a humidified chamber at room temperature for 1 h. After incubation, the specimens were rinsed by immersion in PBS solution, and DAPI-containing Vectashield mounting medium (Vector Laboratories, U.S.) was applied dropwise. The slides were then observed under an inverted confocal fluorescence microscope (Nikon, Japan). The negative control group consisted of specimens processed without the primary antibody.

### Western blot

CFS and levator palpebral muscle specimens from the same eye were removed from the refrigerator, and RIPA lysis buffer (Soleibao Co., China) was added to homogenize and lyse the tissue thoroughly. The lysate was centrifuged at 13,300 rpm for 15 min at 4 °C to collect the supernatant protein solution, which contained the total protein extract of the tissues. A BCA protein quantification kit (Soleibao Co., China) was used to determine the protein concentration. Twenty micrograms of protein per well was subjected to 10% SDS-PAGE electrophoresis and transferred to a PVDF membrane, which was then blocked with 5% skim milk powder blocking solution at room temperature for 1 h. After cutting the PVDF membrane, the rabbit anti-human elastin antibody (1:1000, Abcam, USA) and an internal control anti-actin antibody (1:5000, Unitech Co., China) were added to each membrane to react overnight at 4 °C; the membranes were then washed with TBST solution and incubated with horseradish peroxidase (HRP-conjugated sheep anti-rabbit, anti-goat and anti-mouse IgG secondary antibodies (1:5000, Boster Co., China) at room temperature for 1 h. The membranes were washed with TBST solution, and an enhanced chemiluminescence (ECL) kit (Boster Co., China) was used to develop the protein signals. An image was captured with a gel scanner, and ImageJ software was applied to analyze the gray values of the protein bands. The ratio of the gray value of the target protein band to that of the internal reference protein band represents the relative protein expression.

### Statistical analysis

Statistical analysis was performed using SPSS 20.0 software (SPSS Inc, Chicago, IL, USA). Measurement values are presented as the mean ± standard deviation, and comparisons between multiple groups were performed by one-way *ANOVA*. A *P* value less than 0.05 was considered statistically significant. Illustrator CS6 (Adobe Inc, CA, USA) software was used for drawing.

## Results

### Outcome of CFS + LM suspension

The curative effect six months after CFS + LM suspension is listed in Table 1. In the adult group, the well corrective rate of CFS + LM suspension was 100% at the end of follow-up. Furthermore, there was 1 case of undercorrection each in the adolescent group and the children group, with 88.89% corrective rate. In addition, there were no relapses in the three groups within six months after surgery. Complications following CFS + LM suspension during the whole follow-up in the three groups are listed in Table 2. Lagophthalmos was observed in 2 patients (2 eyes), including 1 patient in the adult group and 1 patient in the child group, and almost disappeared within 6 months postoperatively. Moreover, there were 2 patients (2 eyes) with conjunctival prolapse within 1 week after surgery, including 1 patient in the adult group and 1 patient in the adolescent group. Those patients recovered after the fixation of conjunctiva fornix to the corresponding eyelid with mattress-suture using 4–0 silk threads, which were removed after two weeks. In addition, 1 patient showed mild eyelid hematoma in the child group, which recovered within one month. Other postoperative complications, such as exposure keratitis, separation of eyelid from eyeball, trichiasis, superior sulcus deepening, and abnormal ocular motility did not occur after CFS + LM surgery.

**Table 1:** Corrective effect six months after CFS + LM suspension

Curative effect	Adult group (*n* = 9)	Adolescent group (*n* = 9)	Child group (*n* = 9)
Good correction	9	8	8
Undercorrection	0	1	1
Overcorrection	0	0	0
Relapse	0	0	0
Correction rate	100%	88.89%	88.89%

**Table 2:** Postoperative complications of CFS + LM suspension during follow-up

Complications	Adult group (*n* = 9)	Adolescent group (*n* = 9)	Child group (*n* = 9)
Lagophthalmos	1	0	1
Exposure keratitis	0	0	0
Eyelid hematoma	0	0	1
Conjunctival prolapse	1	1	0
Entropion	0	0	0
Ectropion	0	0	0
Eyelid-ball separation	0	0	0
Eyeball motility restriction	0	0	0
Superior sulcus deepening	0	0	0
**Total complications**	2	1	2

### Histology

**Fig. 2:**
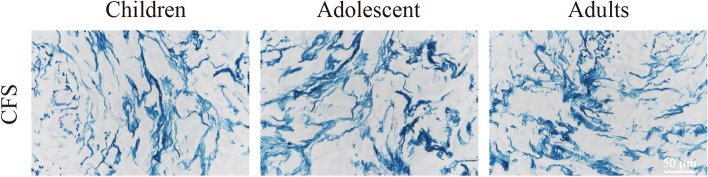
Microscopic images of the CFS sections. (Victoria Blue staining, × 10)

On Victoria Blue staining of elastic fibers, the CFS specimens of the adult group showed an extensive striated pattern of blue staining, confirming that adult CFS tissue was rich in elastic fibers (Fig. 2).

### Immunofluorescence

**Fig. 3:**
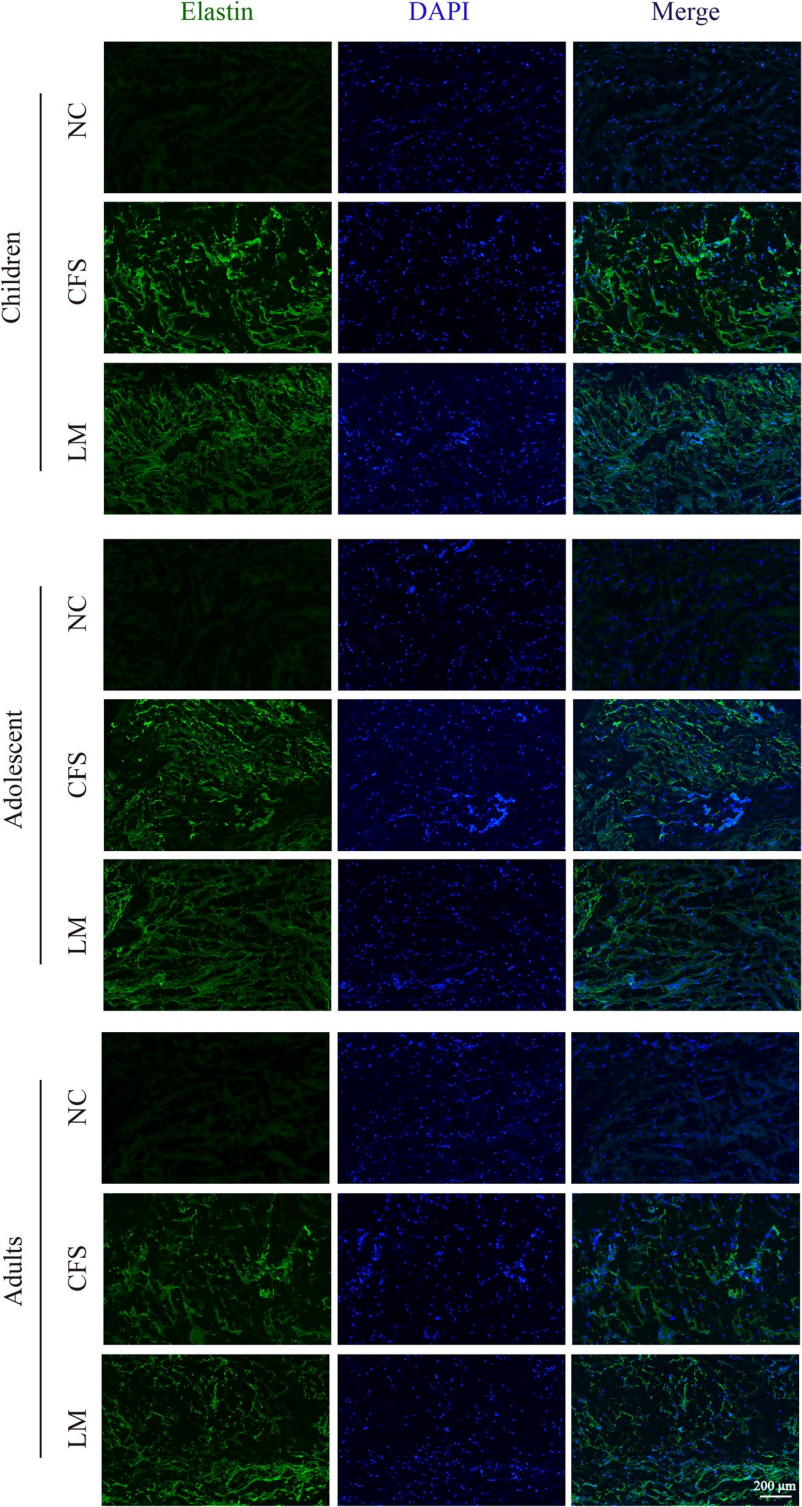
Results of immunofluorescent staining of the CFS and LM in the three age groups. (NC: negative contrast)

Immunofluorescent staining revealed strong positive expression of elastin in the CFS and LM tissues of the adult, adolescent and child groups; the elastin was distributed in an interwoven network (Fig. 3).

### Western blot

**Fig. 4:**
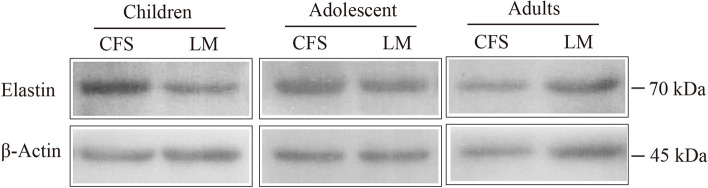
Western blot showing elastin/β-actin expression in the CFS and LM in the three age groups

**Fig. 5:**
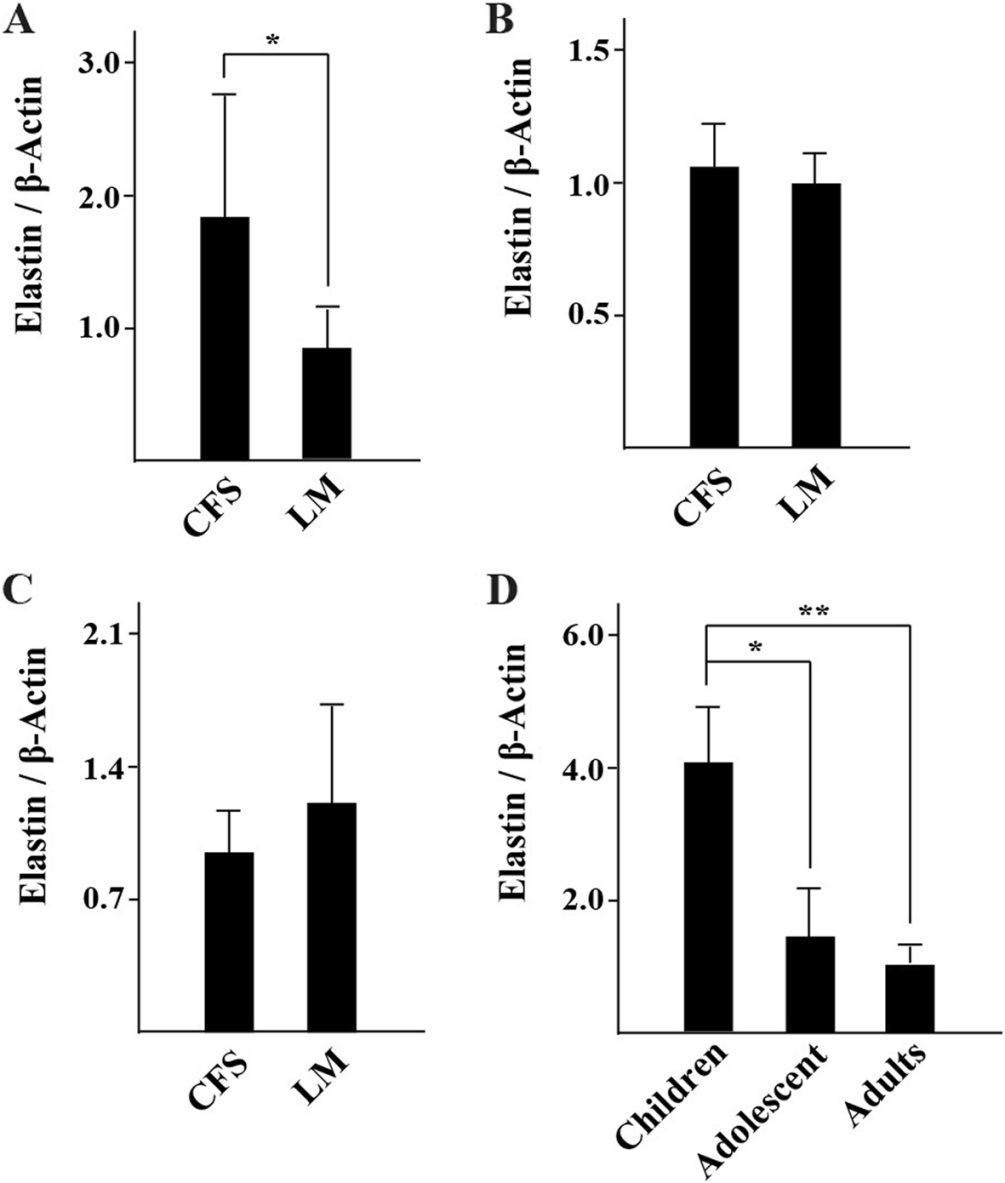
Elastin expression in the CFS and LM of all groups. **A**: child group; **B**: adolescent group; **C**: adult group; **D**: elastin expression of CFS in the child, adolescent and adult groups. (* *P* < 0.05, ** *P* < 0.01)

The Western blot results showed the difference in elastin expression between the CFS and LM (Fig. 4). As can be seen in Table 3, elastin was expressed in both the CFS and LM in all groups. In the adult group, elastin expression, presented as the ratio of elastin to actin, was measured at 0.95 ± 0.22 in the CFS and 1.21 ± 0.53 in the LM; there was no significant difference between these values (*P* > 0.05, Fig. 5B). Similarly, in the adolescent group, elastin expression measured 0.95 ± 0.22 in the CFS and 1.21 ± 0.53 in the LM, and there was no significant difference in elastin expression between these two locations (*P* > 0.05, Fig. 5C). In the child group, however, elastin expression measured 1.83 ± 0.93 in the CFS and 0.83 ± 0.32 in the LM, with the relative expression being significantly higher in the CFS than in the LM (*P* < 0.05, Fig. 5A).

**Table 3:** Comparison of elastin/β-actin expression between the CFS and the LM in each of the three age groups ( $\overline{x }$± s)

Groups	Tissues	n (eyes)	Elastin/β-actin	*P* value
Adults	CFS	9	0.95 ± 0.22	*P* > 0.05
	LM	9	1.21 ± 0.53	
Adolescents	CFS	9	1.05 ± 0.17	*P* > 0.05
	LM	9	0.99 ± 0.11	
Children	CFS	9	1.83 ± 0.93	*P* < 0.05
	LM	9	0.83 ± 0.32

**Fig. 6:**
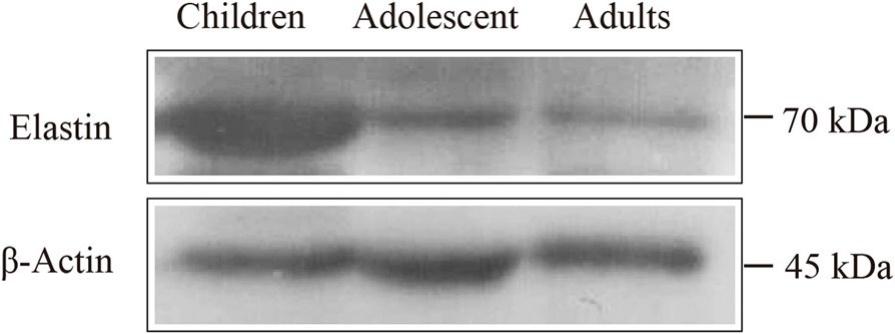
Western blot of elastin expression in the CFS in the child, adolescent and adult groups

As shown in Table 4, elastin expression in the CFS of the child, adolescent and adult groups measured 4.05 ± 0.84, 1.46 ± 0.74 and 1.02 ± 0.24, respectively. Western blot results (Fig. 6) showed that compared with the child group, the adolescent group and the adult group had reduced relative expression of elastin in the CFS, and the difference was statistically significant (*P* < 0.05 and *P* < 0.01, respectively, Fig. 5D). Compared with the adolescent group, the adult group had reduced relative expression of elastin in the CFS, but the difference was not statistically significant (*P* > 0.05, Fig. 5D).

**Table 4:** Elastin expression in the CFS in the adult, adolescent and child groups ($\overline{x }$ ± s)

Groups	n (eyes)	Elastin/β-actin
Adults	9	1.02 ± 0.24
Adolescents	9	1.46 ± 0.74
Children	9	4.05 ± 0.84

## Discussion

Congenital blepharoptosis is characterized by an abnormally low-lying upper eyelid margin at birth, which is caused by dysfunction of the LM or Müller muscle [8]. Blepharoptosis may be minimal (1–2 mm), moderate (3–4 mm), or severe (> 4 mm), covering the pupil entirely [9]. This disease can result in narrowing of the palpebral fissure and visual field [9], and may seriously affect aesthetics and mental health. At present, traditional corrective methods for blepharoptosis can be categorized into 3 classifications [4], such as levator resection, Müller muscle conjunctival resection [10] and frontalis suspension. However, these surgeries are associated with many complications, such as lagophthalmos, corneal exposure, forehead wrinkle aggravation and eyebrow raising [11]. Thus, new surgical techniques are needed to cure severe blepharoptosis.

In the past decade, CFS has attracted a great deal of attention as a cure for congenital blepharoptosis [4, 12]. Much earlier, in 1805, Tenon discovered and described the existence of the CFS, believing it to be the tendinous fascia of the superior rectus muscle; functionally, this connective structure was reported to assist the function of the rectus muscle and promote the movement of the upper eyelid [13]. In 1874, Merkel proposed the concept of the check ligament and applied that name to this tendon fascia [14]. In 1885, Lockwood confirmed that there were check ligaments attached to the superior extraocular rectus muscles [15, 16]. Subsequently, many anatomists and surgeons continued to study this tendon fascia in depth, describing it as the joint fascial sheath of the LM and superior rectus muscle, the expansion of the superior transverse fascia [17], the transverse intermuscular ligament [18], or the check ligament of the superior fornix [13]; their studies revealed that it is more closely connected to the LM than to the superior rectus muscle and has the effect of balancing the contraction function of the LM.

In 2002, Holmström introduced a technique of “check ligament” suspension for blepharoptosis correction [3]. In 2008, Hwang proposed that this structure is a piece of fibrous connective tissue formed by the fusion and proliferation of the LM and superior rectus fascia, naming it the conjoint fascial sheath, or CFS [19]. In recent years, many studies have applied CFS suspension or CFS + LM complex suspension to correct severe congenital blepharoptosis; these procedures have been found to have many advantages [4, 12, 20], such as a lack of obvious separation of the eyelid from the eyeball, a lack of severe postoperative discomfort, a natural double eyelid, swift postoperative recovery and intact eyebrow and forehead tissues. The mechanism of CFS + LM suspension is to suture the CFS + LM complex to the tarsal plate through a double eyelid incision, which lifts the upper eyelid. Compared with CFS suspension alone, the advantage of CFS + LM complex suspension is that it provides additional force to suspend the upper eyelid, which makes its position more stable after the operation and enables a better long-term effect.

In recent, the histological study of the CFS [1] and LM [21] has received much attention among ophthalmologists. In our study, the histological results showed that the CFS is a fibrous connective tissue membrane. In line with Bei and Yang’s study, Victoria Blue staining showed that the CFS contained a large number of flexible elastic fibers and collagen fibers, which may explain why the CFS is elastic and why CFS surgery can sometimes even replace frontalis suspension for severe blepharoptosis [1].

Elastin is the most important structural component of elastic fibers [6]. For the first time, this study investigated the expression of elastin in the CFS and LM. The results of immunofluorescent staining showed that the CFS and LM tissues of patients with severe blepharoptosis contained strong positive expression of elastin, which was distributed in an intertwined network. In addition, a Western blot and semiquantitative analysis of elastin expression were performed in the adult, adolescent and child groups. The experimental results showed that there was no significant difference in elastin expression between the CFS and the LM in either adults or adolescents (*P* > 0.05). However, in the child group, the expression of elastin was significantly higher in the CFS than in the LM (*P* < 0.05). This result suggests that the LM and CFS of eyes with severe congenital blepharoptosis are rich in elastic fibers and have high elastin expression, which implies that clinicians can apply CFS + LM complex suspension surgery to correct severe congenital blepharoptosis. In addition, further analysis of the expression of elastin in the CFS of adults, adolescents and children showed that the CFS tissue of adults and adolescents had significantly lower elastin expression than that of children (*P* < 0.05), indicating that elastin expression in the CFS decreased with age. These results support the feasibility of CFS + LM suspension surgery as a cure for severe congenital blepharoptosis in pediatric patients.

The limitations of the present study must also be acknowledged. First, a larger sample size is needed in future studies. Second, the relationship between elastin expression and the long-term postoperative effect of CFS + LM suspension needs to be investigated. Third, the elastin expression of LM in different age groups needs to be explored. In summary, our study confirmed that the CFS and LM are rich in elastic fibers and elastin; additionally, this study tested the elastin expression of the CFS in different age groups for the first time, demonstrating that elastin expression in the CFS decreases with age. Thus, it is feasible to apply CFS + LM complex suspension surgery to cure severe congenital blepharoptosis in pediatric patients.

## Supplementary Information

**Additional file 1.** Original Western blot Gels.

## Data Availability

All data are available from the corresponding author upon reasonable request.

## Abbreviations

CFS: Conjoint Facial Sheath
LM: Levator Palpebrae Muscle
MRD1: Margin Reflex Distance 1
OCT: Optimal Cutting Temperature
SDS-PAGE: Sodium Dodecyl Sulfate—Polyacrylamide Gel Electrophoresis

## References

[1] Li B, Yang J, Wu W, Chai C, Gu Z, He Z, Tan Z, Cheng S, Lu P, Zeng L (2020). Anatomical and Histological Study of the Conjoint Fascial Sheath of the Levator and Superior Rectus for Ptosis Surgery. Ophthalmic Plast Reconstr Surg.

[2] Holmstrom H, Bernstrom-Lundberg C, Oldfors A (2002). Anatomical study of the structures at the roof of the orbit with special reference to the check ligament of the superior fornix. Scand J Plast Reconstr Surg Hand Surg.

[3] Holmstrom H, Santanelli F (2002). Suspension of the eyelid to the check ligament of the superior fornix for congenital blepharoptosis. Scand J Plast Reconstr Surg Hand Surg.

[4] Xing Y, Wang X, Cao Y, Ding X, Lin M, Li J, Fan X (2019). Modified Combined Fascia Sheath and Levator Muscle Complex Suspension With Muller Muscle Preservation on Treating Severe Congenital Ptosis. Ann Plast Surg.

[5] Li Y, Wang H, Bai P (2021). Changes of Ocular Surface Before and After Treatment of Blepharoptosis With Combined Fascial Sheath Suspension and Frontal Muscle Flap Suspension. J Craniofac Surg.

[6] Mithieux SM, Weiss AS (2005). Elastin. Adv Protein Chem.

[7] Zuo L, Wang XX, Huang XY, Zhang JL, Du YY (2017). A Modified Levator Resection Technique Involving Retention of the Levator Palpebrae Superioris Muscle Suspension System for Treatment of Congenital Ptosis. Aesthetic Plast Surg.

[8] Patel K, Carballo S, Thompson L (2017). Ptosis. Dis Mon.

[9] Finsterer J (2003). Ptosis: causes, presentation, and management. Aesthetic Plast Surg.

[10] Mazow ML, Shulkin ZA. Mueller’s muscle-conjunctival resection in the treatment of congenital ptosis. Ophthalmic Plast Reconstr Surg. 2011;27(5):311–2. 10.1097/IOP.0b013e31820d8749 21415804

[11] Massry GG (2005). Ptosis repair for the cosmetic surgeon. Facial Plast Surg Clin North Am.

[12] Wang H, Liu L, Wang ZJ (2020). Conjoint fascial sheath suspension for early correction of severe blepharoptosis after double-eyelid blepharoplasty. Br J Oral Maxillofac Surg.

[13] Nam YS, Kim IB, Shin SY (2015). Detailed anatomy of the transverse superior fascial expansion of the upper eyelid. Graefes Arch Clin Exp Ophthalmol.

[14] Whitnall SE (1911). A Ligament acting as a Check to the Action of the Levator Palpebrae Superioris Muscle. J Anat Physiol.

[15] Manson PN, Clifford CM, Su CT, Iliff NT, Morgan R (1986). Mechanisms of global support and posttraumatic enophthalmos: I. The anatomy of the ligament sling and its relation to intramuscular cone orbital fat. Plast Reconstr Surg.

[16] Lockwood CB. The Anatomy of the Muscles, Ligaments, and Fasclae of the Orbit, including an Account of the Capsule of Tenon, the Check Ligaments of the Recti, and the Suspensory Ligaments of the Eye. J Anat Physiol. 1885;20(Pt 1):i2–25. PMC1288533 17231613

[17] Wh FINK (1957). An anatomic study of the check mechanism of the vertical muscles of the eyes. AM J OPHTHALMOL.

[18] Lukas JR, Priglinger S, Denk M, Mayr R. Two fibromuscular transverse ligaments related to the levator palpebrae superioris: Whitnall’s ligament and an intermuscular transverse ligament. Anat Rec. 1996;246(3):415–22. 10.1002/(SICI)1097-0185(199611)246:3<415::AID-AR13>3.0.CO;2-R 8915464

[19] Hwang K, Shin YH, Kim DJ (2008). Conjoint fascial sheath of the levator and superior rectus attached to the conjunctival fornix. J Craniofac Surg.

[20] Zhou J, Chen W, Qi Z, Jin X (2019). Minimally Invasive Conjoint Fascial Sheath Suspension for Blepharoptosis Correction. Aesthetic Plast Surg.

[21] Quaranta-Leoni FM, Secondi R, Quaranta-Leoni F, Nardoni S (2020). Histological findings of levator muscle in unilateral congenital ptosis in different age groups. Acta Ophthalmol.

