# Supplementary material for: Research on the expression of elastin in the conjoint fascial sheath for the correction of severe unilateral congenital blepharoptosis
Source: BMC Ophthalmol. 2022 Jun 8;22:256. doi: 10.1186/s12886-022-02469-w (PMC9175472; doi:10.1186/s12886-022-02469-w)
Supplement: Supplementary file 1 — Additional file 1. Original Western blot Gels. [file 12886_2022_2469_MOESM1_ESM.docx]

**Original Western blot Gels**

(Title：Research on the Expression of Elastin in the Conjoint Fascial Sheath for the Correction of Severe Unilateral Congenital Blepharoptosis)

1. **Western blot gels in Figure 4 （child group）**
   1. **Elastin:**


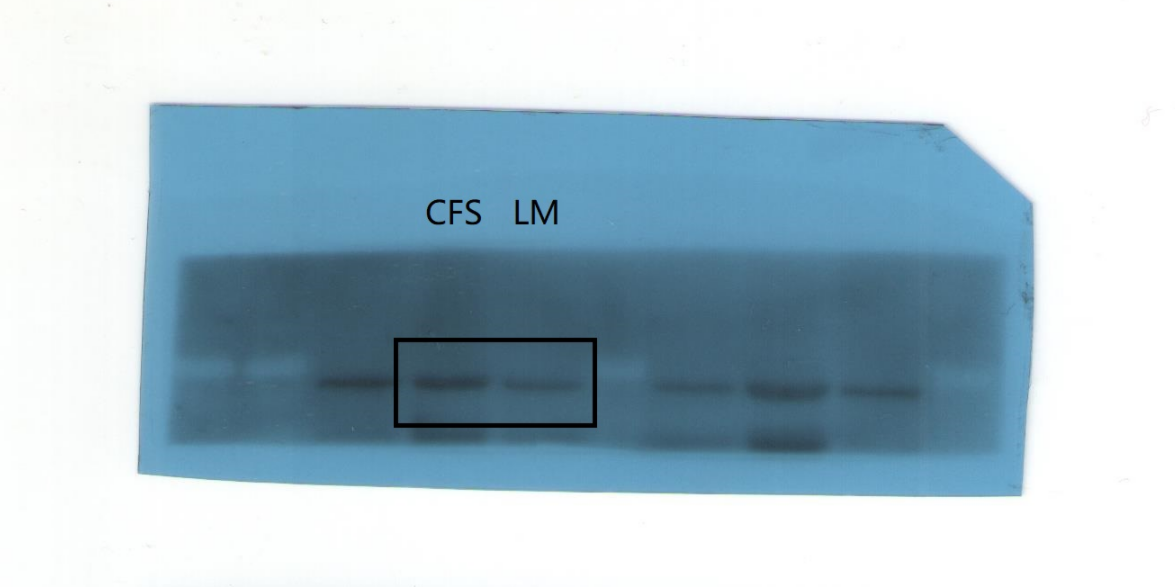


**75 kDa**

Western blot result in Fig.4 (elastin expression of CFS and LM in child group)

**1.2 Beta-actin:**


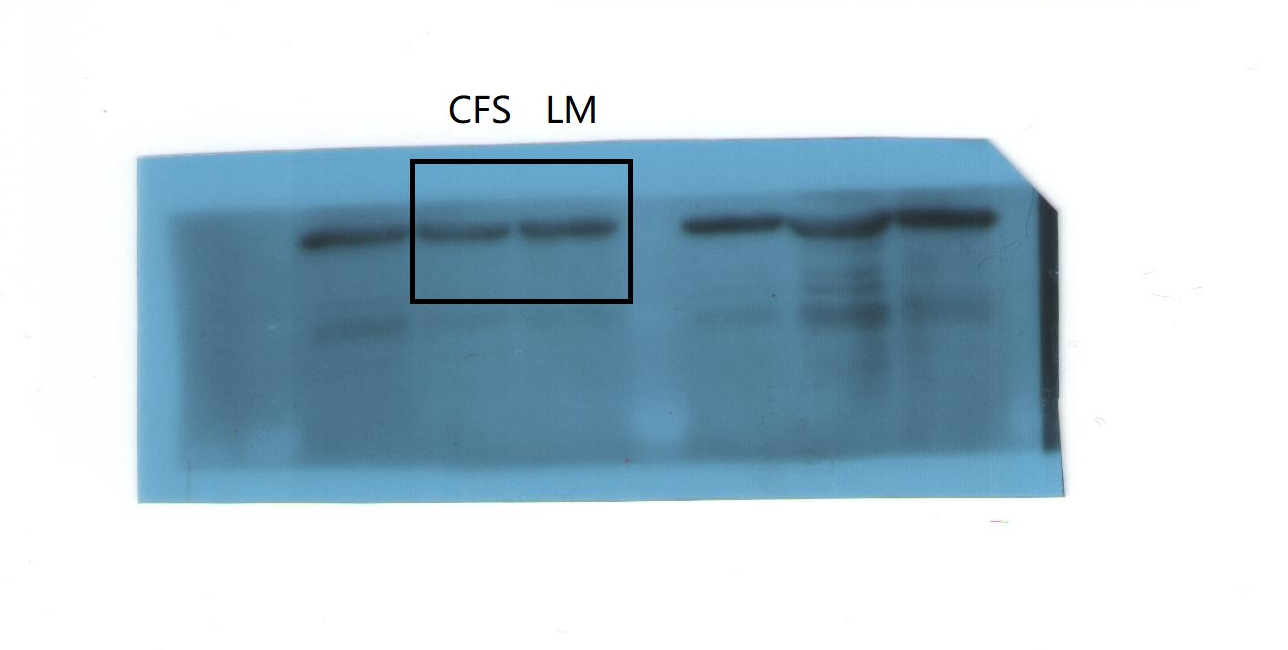


**48 kDa**

Western blot result in Fig.4 (β-actin expression of CFS and LM in child group)

**1.3 Marker:**


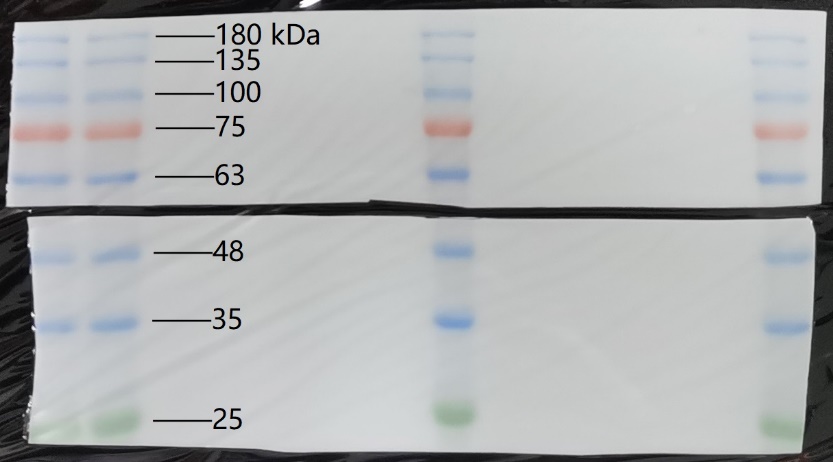


PVDF membrane of western blot result in Fig.4 (elastin and β-actin expression of CFS and LM in children group)

**2 Figure 4 （adolescent group）**

**2.1 Elastin:**


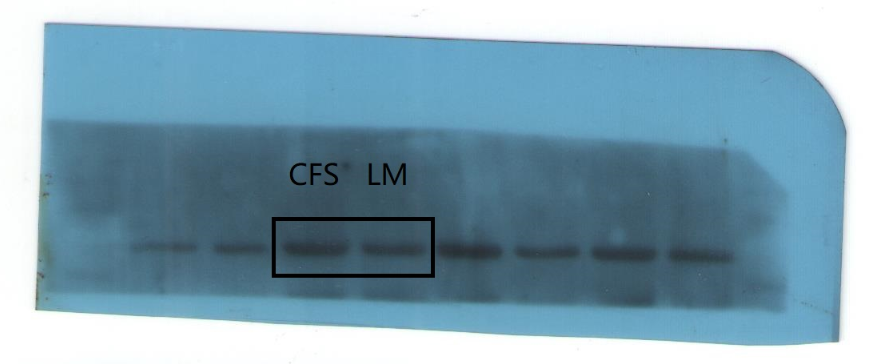


**75 kDa**

Western blot result in Fig.4 (elastin expression of CFS and LM in adolescent group)

**2.2 beta-actin:**


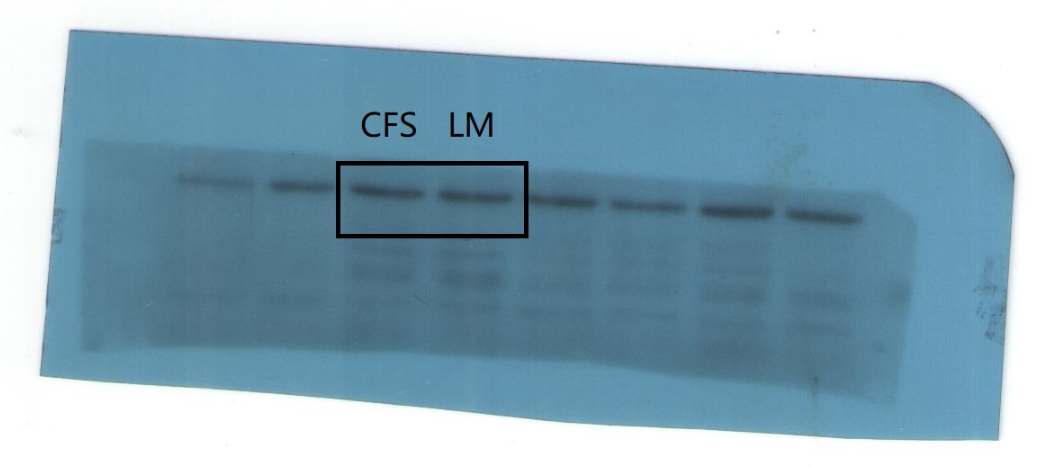


**48 kDa**

Western blot result in Fig.4 (β-actin expression of CFS and LM in adolescent group)

**2.3 Marker:**


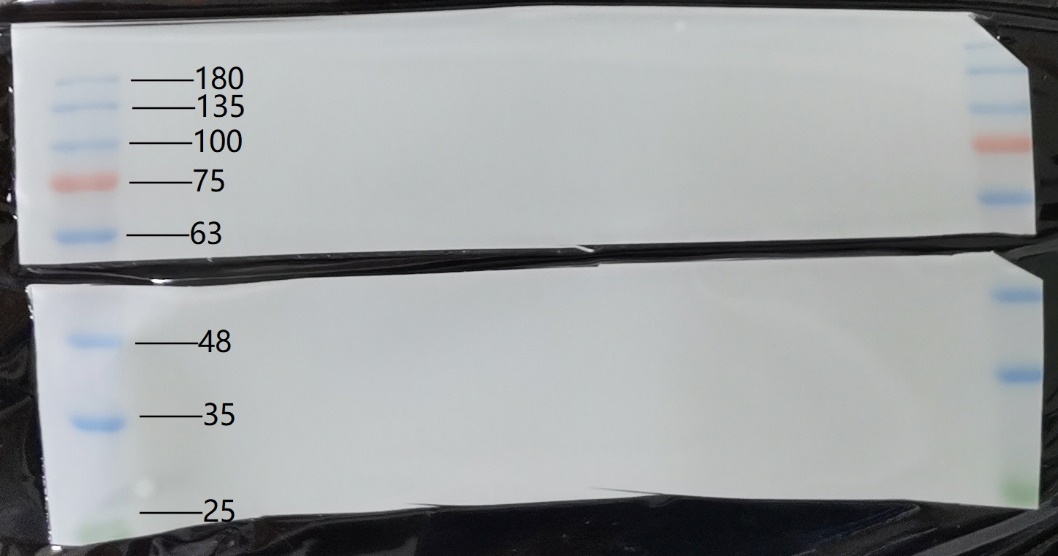


PVDF membrane of western blot result in Fig.4 (elastin and β-actin expression of CFS and LM in adolescent group)

**3） Figure 4 （adult group）**

**3.1 Elastin:**


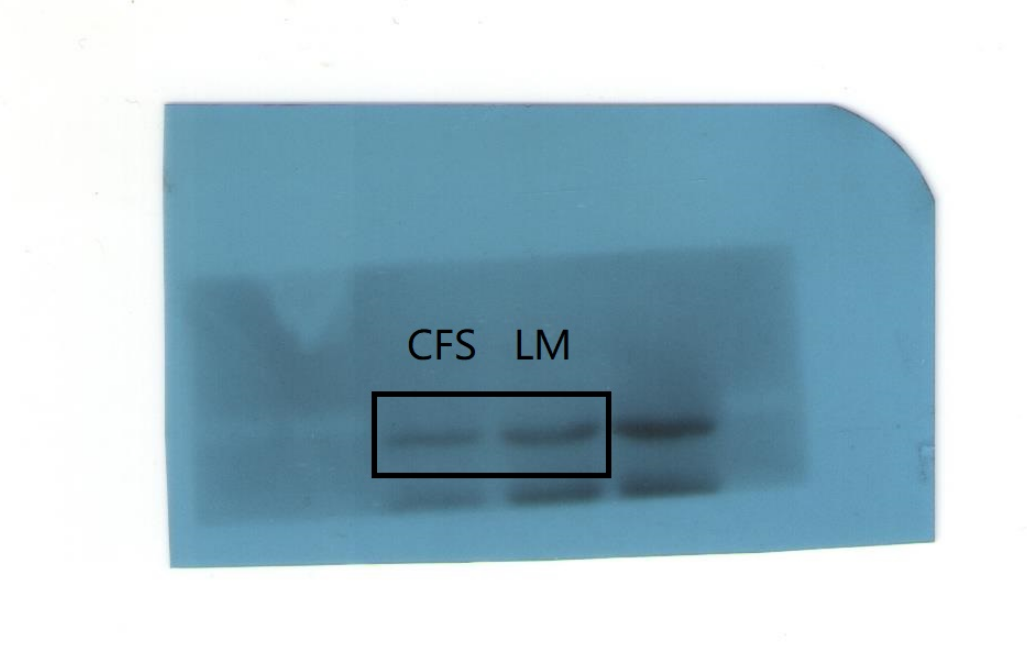


**75 kDa**

Western blot result in Fig.4 (elastin expression of CFS and LM in adult group)

**3.2 beta-actin:**


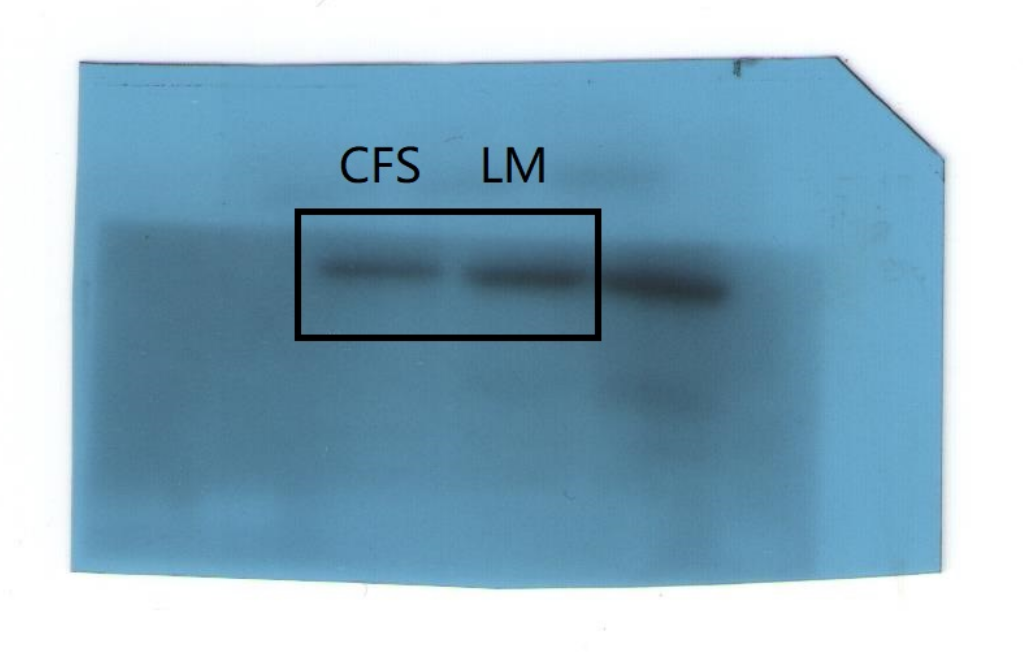


**48 kDa**

Western blot result in Fig.4 (β-actin expression of CFS and LM in adult group)

**3.3 Marker:**


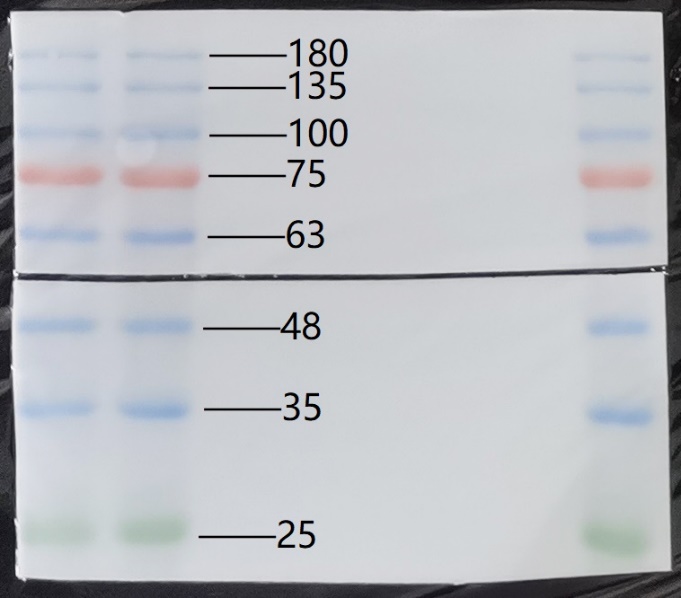


PVDF membrane of western blot result in Fig.4 (elastin and β-actin expression of CFS and LM in adult group)

1. **Figure 6**

**4.1 Elastin:**


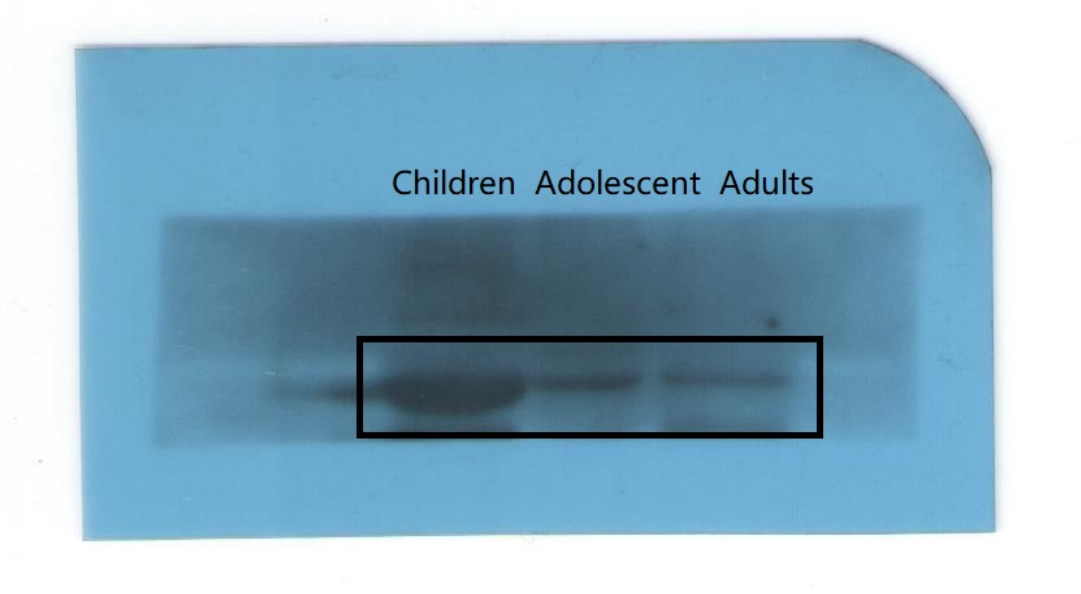


**75 kDa**

Western blot result in Fig.6 (Elastin expression in three different age groups)

**4.2 beta-actin:**


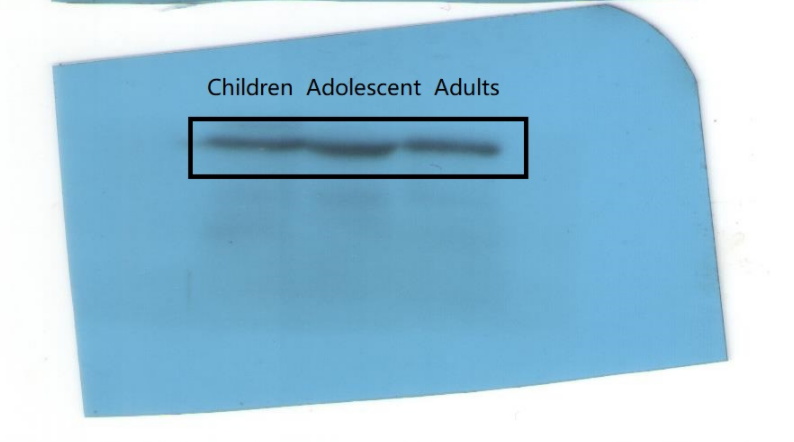


**48 kDa**

Western blot result in Fig.6 (beta-actin expression in three different age groups)

**4.3 Marker:**


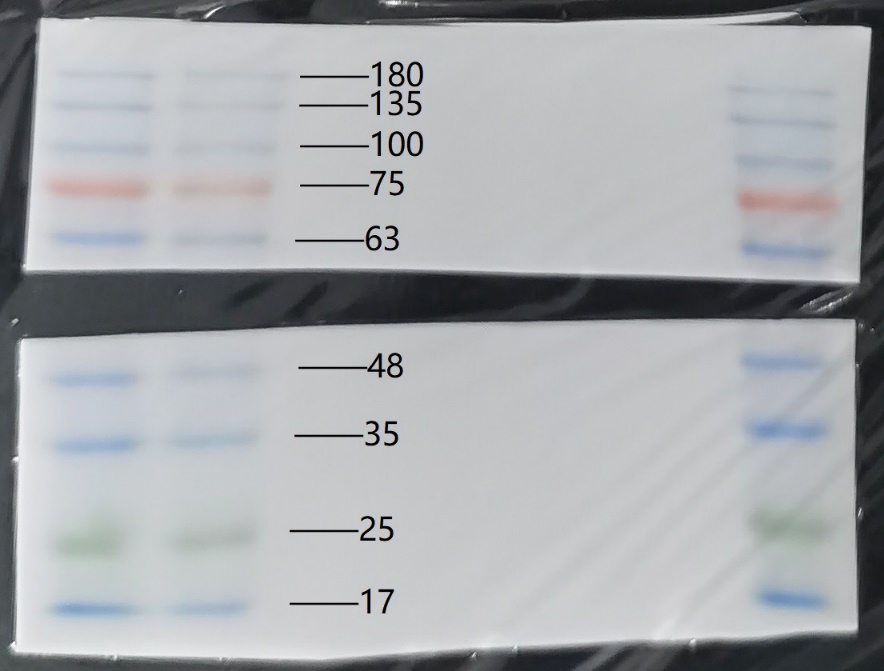


PVDF membrane of western blot result in Fig.6 (elastin and β-actin expression of CFS in three different age groups)
